# Novel Role for PD-1:PD-L1 as Mediator of Pulmonary Vascular Endothelial Cell Functions in Pathogenesis of Indirect ARDS in Mice

**DOI:** 10.3389/fimmu.2018.03030

**Published:** 2018-12-20

**Authors:** Joanne Lomas-Neira, Sean F. Monaghan, Xin Huang, Eleanor A. Fallon, Chun-Shiang Chung, Alfred Ayala

**Affiliations:** Division of Surgical Research, Department of Surgery, Rhode Island Hospital/Alpert School of Medicine at Brown University, Providence, RI, United States

**Keywords:** Angiopoietin-1 and-2, Tie2, lung permeability, innate immunity, lung injury

## Abstract

Deficiency of the co-inhibitory receptor, Programmed cell death receptor (PD)-1, provides a survival benefit in our murine shock/sepsis model for the development of indirect acute respiratory distress syndrome (iARDS). Further, of clinical significance, patients that develop ARDS express increased PD-1 on their blood leukocytes. While PD-1 expression and its regulatory role have been associated with mainly T-cell responses, the contribution of its primary ligand, PD-L1, broadly expressed on non-immune cells such as lung endothelial cells (ECs) as well as immune cells, is less well-understood. Here we show that a “priming insult” for iARDS, such as non-lethal hemorrhagic shock alone, produced a marked increase in lung EC PD-L1 as well as blood leukocyte PD-1 expression, and when combined with a subsequent “trigger event” (polymicrobial sepsis), not only induced marked iARDS but significant mortality. These sequelae were both attenuated in the absence of PD-L1. Interestingly, we found that gene deficiency of both PD-1 and PD-L1 improved EC barrier function, as measured by decreased bronchoalveolar lavage fluid protein (i.e., lung leak). However, PD-L1 deficiency, unlike PD-1, significantly decreased EC activation through the Angiopoietin/Tie2 pathway in our iARDS mice. Additionally, while PD-1 gene deficiency was associated with decreased neutrophil influx in our iARDS mice, EC monolayers derived from PD-L1 deficient mice showed increased expression of EC junction proteins in response to *ex vivo* TNF-α stimulation. Together, these data suggest that ligation of PD-1:PD-L1 may play a novel role(s) in the maintenance of pulmonary EC barrier regulation, beyond that of the classic regulation of the leukocyte tolerogenic immune response, which may account for its pathogenic actions in iARDS.

## Key Messages

- PD-L1 gene deficiency imparts survival benefit to mice with indirect ARDS
- In ARDS mice, loss of PD-L1 signaling reduces EC Ang-2 release and contributes to restoration of pericyte Ang-1 release
- Hemorrhagic shock increases percentage of PD-1^+^ neutrophils in blood and PD-L1^+^ endothelial cells in lung
- PD-L1 gene deficiency decreases EC activation/lung tissue injury (maintains EC junctional integrity)
- Neutrophil expression of PD-L1 is potentiated following influx to lung in indirect ARDS

## Introduction

Programmed cell death receptor (PD)-1 and its ligand, PD-L1 (B7H1), are primarily known for their roles as negative regulatory molecules modulating leukocyte activation and sustaining antigen tolerance (1). PD-1 is broadly expressed in lymphoid tissue and organs (2–4), and its primary ligand, PD-L1, has been shown, additionally, to be widely expressed on a variety of immune and non-immune cells including lung vascular endothelial cells (ECs) (5).

While many current studies focus on the PD-1:PD-L1 pathway in T-cell immunoregulatory responses, our laboratory has demonstrated that expression of PD-1 contributes to mortality in our murine model of hemorrhagic shock (Hem) priming for the development of indirect acute respiratory distress syndrome (iARDS) following a subsequent septic challenge (CLP) (6, 7). This finding is of specific clinical interest since we have previously reported data showing that trauma intensive care unit (TICU) patients with lower levels of PD-1^+^ blood leukocytes have a greater likelihood of surviving ARDS (8). We have also reported increased PD-1 expression on Ly6G^+^/neutrophils in our murine sequential hemorrhagic shock/polymicrobial septic challenge (cecal ligation & puncture [CLP]) model for the development of iARDS (4). For this study, further investigating the interactions between neutrophils and pulmonary vascular ECs in the pathogenesis of iARDS, we have chosen to focus on the impact of PD-L1 or PD-1 deficiency on these cells.

ARDS is a complication of trauma characterized by increased microvascular permeability, pulmonary edema, inflammation, and neutrophil accumulation in the lung. Advances in supportive care have reduced mortality associated with ARDS (9), however, morbidity and long-term survival remain a major concern (10, 11). ARDS can stem from either direct or indirect/extra-pulmonary factors; direct ARDS includes pneumonia, aspiration and lung trauma, while indirect (i) ARDS is associated with extra-pulmonary sepsis and multisystem trauma (12–14). The complexity of these multi-systemic factors has likely contributed to the pathogenesis of iARDS being less well-understood. To this end, we have investigated a number of proteins associated with the pathogenesis of iARDS in our murine model (4, 15–17).

Inhibition of neutrophil migration (via antibody and siRNA blockade of chemokines MIP-2 & KC) decreases indices of iARDS (15) and EC interaction with hemorrhage-primed neutrophils plays a significant role in EC activation and release of Angiopoietin-2 (Ang-2). Ang-2 is an EC growth factor that plays a significant role in EC loss of function (i.e., edema/increased microvascular permeability) in the development of iARDS (16).

The extent to which EC PD-L1 expression and/or EC interaction with PD-1 expressing neutrophils contributes to lung EC dysfunction encountered during iARDS has not yet been determined. In this study we investigated the role of co-inhibitory molecule, PD-L1, as well as the potential contribution of PD-1:PD-L1 interactions, in the development of iARDS in mice following hemorrhagic shock and subsequent septic challenge.

## Methods

### Mice

Male C57BL/6J mice (Jackson Laboratories, Bar Harbor, ME) were used for wild-type control (WT). B7-H1/PD-L1 (PD-L1^−/−^) (18) and PD-1^−/−^ gene deficient mice were generously provided (see acknowledgments) and colonies maintained in our animal care facility. All mice used in this study were 7–11 weeks old. Experiments were performed in accordance with NIH *Guidelines for Animal Use and Care* and approval from Rhode Island Hospital Institutional Animal Care and Use Committee (Providence, RI; AWC# 0125-13 & 0040-16).

### Reagents

Antibodies for PD-1, PD-L1 (B7-H1), Ly6G, ICAM-1, CD31, VE-Cadherin and MIP-2 were obtained from R&D Systems, Minneapolis, MN; anti-CD31 ELISA kit and mouse CBA cytokine assay kit (IL-6, TNF-α, MCP-1) from BD Bioscience, San Diego, CA; and Ang-1 and Ang-2 cytokine assay kits from Life Sciences Advanced Technologies, St. Petersburg, FL. Tie2 and phosphorylated Tie2 ELISA kits purchased from R&D Systems, Minneapolis, MN. All other chemicals were analytical reagent grade.

### Experimental Model of iARDS

A mouse model of hemorrhagic shock induced “priming” followed by the induction of sepsis for the development of iARDS [a model with which we have considerable experience and in which we have shown induces arterial PO_2_/FIO_2_, (mmHg) of ~150 mmHg by 24 h post-shock/sepsis along with evidence of protein leak and morphological changes (19)] was used here (15, 16, 20). Mortality associated with this model is typically 60–70% after 48 h post-sepsis (15, 20). *Hemorrhage (Hem):* As previously described (15, 20), we used a 90-min, fixed pressure (35 ± 5 mmHg) Hem followed by resuscitation with Ringers lactate at 4X drawn blood volume.

### Polymicrobial Sepsis (CLP)

Twenty-four post-Hem, sepsis was induced as a secondary challenge via cecal ligation and puncture (CLP) as previously described (15, 20). Timing of this secondary insult is based on our previous findings that hemorrhage “priming” followed 24 H later by CLP produced significantly higher levels of lung pro-inflammatory cytokines, increased myeloperoxidase (MPO) activity, and levels of neutrophil specific chemokine, MIP-2, than when sepsis was induced at later time points (15, 20). Mice were euthanized 24 H post CLP, and lung tissue and blood was harvested.

**Figure 1:**
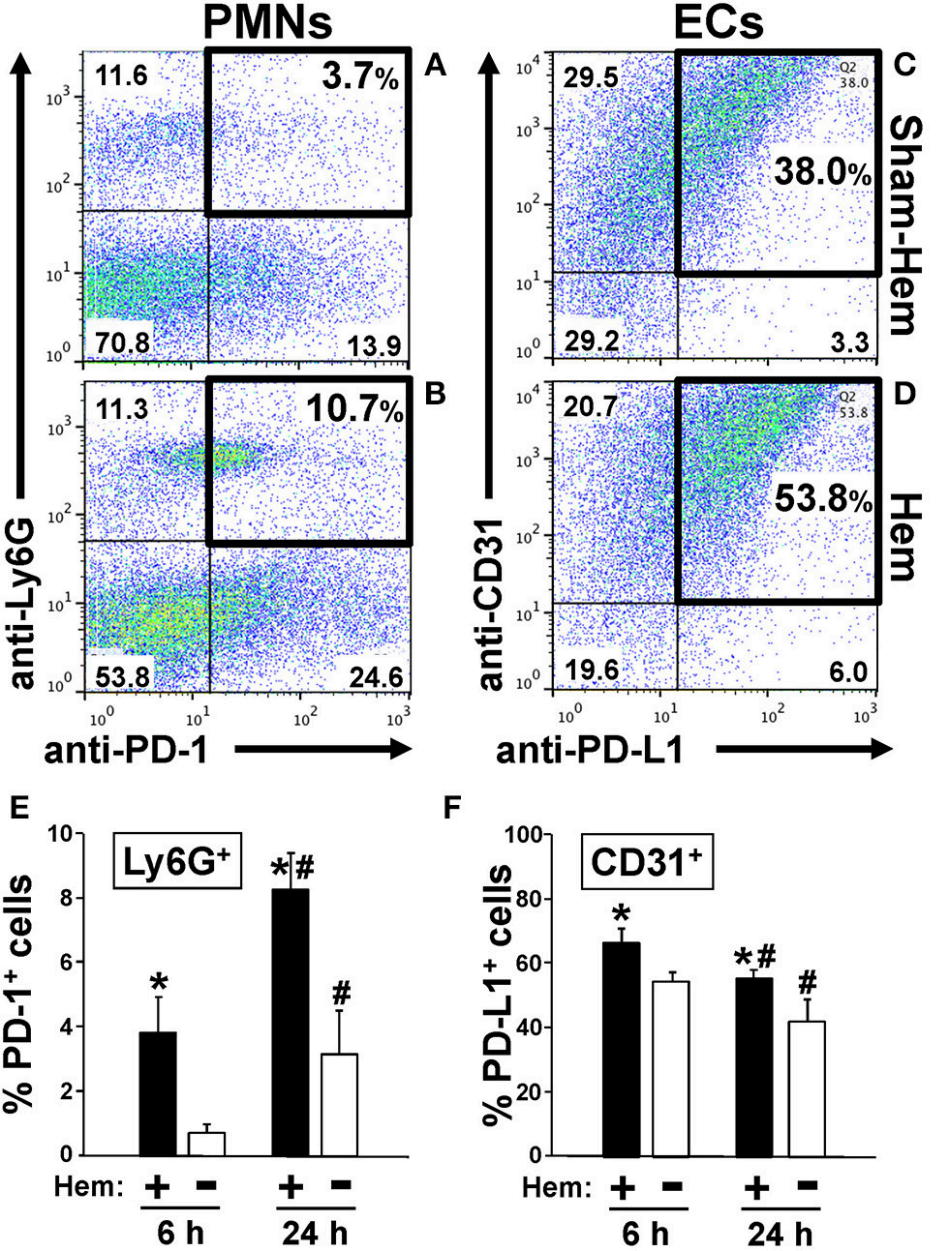
Representative dot-plots illustrating how hemorrhagic shock increased the percentage of PD-1^+^ Ly6G^+^ neutrophils in peripheral blood **(A,B)** and PD-L1^+^ CD31^+^ endothelial cells in lung tissue **(C,D)**. Summary data document that percentage of PD-1^+^ Ly6G^+^ neutrophils increases early and significantly at 6 h and continues to increase over the 24 h following Hem **(E)**. In contrast, the percentage PD-L1^+^ CD31^+^ endothelial cells increase at 6 h post-Hem but decreases by 24 h post-Hem **(F)**. Mean ± SEM, *N* = 3/group, *p < 0.05 vs. Sham Hem; ^#^*p* < 0.05 vs. 6 h. One-way ANOVA and a Tukey's multiple comparisons test.

Inasmuch; except for the initial studies presented in Figure 1 where animals were either subjected to hemorrhage alone (Hem) or a sham protocol (Sham-Hem); WT background control mice, PD-1^−/−^, and/or PD-L1^−/−^ gene deficient mice were randomized to either Hem/CLP (H/C) or the Sham Hem/CLP (SH/C) group.

### Survival Study

iARDS was induced in WT and PD-L1^−/−^ mice as described above. Mice were given access to food and water and assessed for survival over 10-days (2, 21, 22).

### Broncho-Alveolar Lavage Fluid (BALF)

BALF was collected to assess protein concentration as an index of pulmonary vascular permeability (injury) as previously described (20).

### Flow Cytometry (FACS)

Neutrophil and EC expression of PD-L1 or PD-1 was analyzed at 6 or 12 h following Hem by flow cytometry of whole blood and single-cell suspensions of saline-perfused lung tissue as previously described (3, 4, 15, 23, 24). Based on forward/side scatter results for lung ECs, the granulocyte population was gated out. Neutrophils were characterized as Ly6G^+^. Expression of PD-1 or PD-L1 (antibody labeled in whole blood) vs. dissociated antibody labeled lung tissue was calculated as %PD-1^+^Ly6G^+^ and %PD-L1^+^Ly6G^+^, and CD31^+^ (endothelial cells) in lung tissue, and were determined using a FACSarray flow cytometer (BD Bioscience, San Jose, CA). Fluorochrome conjugated antibodies used: anti-Ly6G (clone 1A8), anti-CD-31 (clone 390), anti-B7-H1 (clone MIH5), anti-PD-1 (clone J43). Single cell suspensions of lung tissue from WT control, PD-1^−/−^, and PD-L1^−/−^ mice following SH/C or H/C were also stained to assess EC expression of intercellular adhesion molecule (ICAM)-1. Cells were incubated with PE-Cy7 labeled rat anti-mouse CD31 and BV421 labeled hamster anti-mouse ICAM-1 (Miltenyi biotec).

### Cytokine/Chemokine Assays

Cytokine/Chemokine Assays on lung tissue homogenates, plasma, BALF and culture supernatants were performed as per manufacturer's protocol.

### Myeloperoxidase (MPO) Activity

To assess neutrophil influx to lung, MPO activity in lung tissue homogenates was measured as previously described (20, 25).

### Mouse Lung Endothelial Cell Isolation for Culture

Per experiment, for each group, lungs from 5 mice were enzymatically digested using Miltenyi Biotec (Auburn, CA) mouse lung dissociation kit and GentleMACs™ Dissociator protocols. Cells were passed through a 70 μm screen, washed and incubated with mouse CD45 microbeads. CD45^+^ cells (leukocytes) were depleted using a MACS column and magnetic field. CD45^−^ cells/flow through cells were labeled with mouse CD31 microbeads. CD31^+^ cells were enriched on a MACS column and magnetic field then eluted from the column (Miltenyi Biotec). Cells were washed and resuspended in MCDB131 enriched media (Vec Technologies, Rensselaer, NY), counted, plated onto gelatin-coated six-well plates and grown to confluence. These cells were then used for following different experiments. (1) Cells were stimulated with TNF-α and assessed the changes in VE-Cadherin by Western Blot, and changes in VE-Cadherin/CD31 expression using fluorescent microscopy. Supernatants were collected, spun down, and assessed for Ang-2 and cytokine release. (2) Culture cells from six-well plates were trypsinized and plated onto collagen coated glass coverslips to characterize by fluorescence for von Willebrand factor, acetylated LDL uptake, and CD31 by immunofluorescent staining. (3) Culture cells were trypsinized and plated on Lab-Tek Permanox chambered (8 well) slides at 50,000 cells/per well/treatment in duplicate, 3 separate slides per experiment. When confluent, cells were stimulated with TNF-α for 4 h and assessed for changes in CD31/ICAM-1 expression using flow cytometry (FlowJo, Ashland, OR).

### Fluorescence Microscopy

ECs isolated (as described above) from either WT or PD-L1^−/−^ mouse lungs were grown to confluence on Lab Tek 8 chambered (Permonox) slides coated with 30 ug/ml fibronectin. EC monolayers were treated with either TNF-α (40 ug/ml) or PBS for 4 h. Cells were fixed, permeabilized and blocked (26) prior to dual staining for ICAM-1 (Alexa 647)/ VE-Cadherin (Alexa 488) and VE-Cadherin (Alexa 488)/ ZO-1 (phycoerythrin-PE). Images were captured with a Nikon Eclipse Ni microscope with an ANDOR Zysa sCMOS camera using NIS-Elements BR software.

### Western Blot Analysis

CD31, VE-Cadherin, and ZO-1 protein levels from EC monolayer lysates following TNF-α stimulation was performed as previously described (27)

### Statistical Analysis

Data are expressed as means ± SEM. Statistically significant differences were determined using OneWay ANOVA, SigmaStat v2.03 (*post-hoc* test was Tukey's). Group means were considered significantly different when *p*-values were < 0.05. For survival study, statistical significance was assessed by Kaplan-Meier Survival analysis and comparisons were performed by Chi-square test on each of days post-CLP.

## Results

### PD-1 Expression on Lung Ly6G^+^/Neutrophils Increases Following Hemorrhagic Shock

We have previously reported that expression of PD-1 on Ly6G^+^/neutrophils increased in mouse lungs following iARDS (4). Here we show, both in a typical animal's dissociated lung-tissue flow cytometry dot-plot (Figures 1A,B) and as summary data of repeated experiments (Figure 1E), that 24 h following hemorrhage alone (Hem), the percentage of PD-1^+^ Ly6G^+^/lung tissue neutrophils increased by 35% compared to Sham-Hem. This increase is evident as early as 6 h post-Hem (Figure 1E). Of note, while not the focus of this study we also observed a significant increase in the number of Ly6G^−^ PD-1^+^ sub-population. In this respect, we have previously reported that within this Ly6G^−^ PD-1^+^ sub-population that we could detect CD4^+^ PD-1^+^, CD11c^+^ PD-1^+^ but not CD115^+^PD-1^+^, B220^+^PD-1^+^ nor CD31^+^PD-1^+^ cells in the lungs of iARDS mice (4).

### Hemorrhagic Shock Upregulates the Expression of PD-L1 on Lung Vascular Endothelial Cells

Flow cytometric analysis of dissociated lung tissue cells 24 h following Hem indicates that PD-L1 expression increased on CD31^+^/ECs (Figures 1C,D). At 24H, PD-L1 expression on lung ECs was decreased from 6 h measurement, but still elevated when compared to Sham-Hem (Figure 1F). This 24 h post-Hem time point is significant since, in our model, this is the time point at which sepsis (CLP), as a secondary challenge is induced for the development of iARDS. To further investigate the contribution of increased PD-L1 expression on lung vascular ECs, we assessed the development of iARDS in our model using PD-L1 gene deficient mice (PD-L1^−/−^) compared to WT background control animals and PD-1 deficient (PD-1^−/−^) mice (4).

### PD-L1 Gene Deficiency Imparts a Survival Benefit to Mice With iARDS

**Figure 2:**
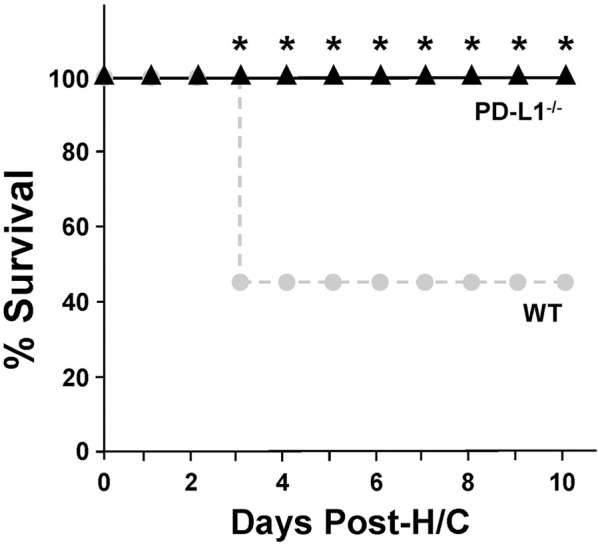
Ten-day percentage (%) survival in PD-L1^−/−^ mice (triangles, *n* = 12) compared to WT (circles, *n* = 13) following H/C. *p < 0.05 vs. WT. Kaplan Meier Survival analysis and comparisons were performed by Chi-square test on each of 10 days post-CLP.

Mice lacking PD-L1 gene expression [much like we previously reported for PD-1 ^−/−^ mice (4)] exhibit a significant survival benefit following H/C iARDS compared to WT controls. A 45% survival benefit was seen at day 3 for PD-L1^−/−^ mice and this was sustained until euthanasia on day 10 (Figure 2).

### Both PD-1 and PD-L1 Gene Deficiency Is Associated With Reduced Lung Injury

**Figure 3:**
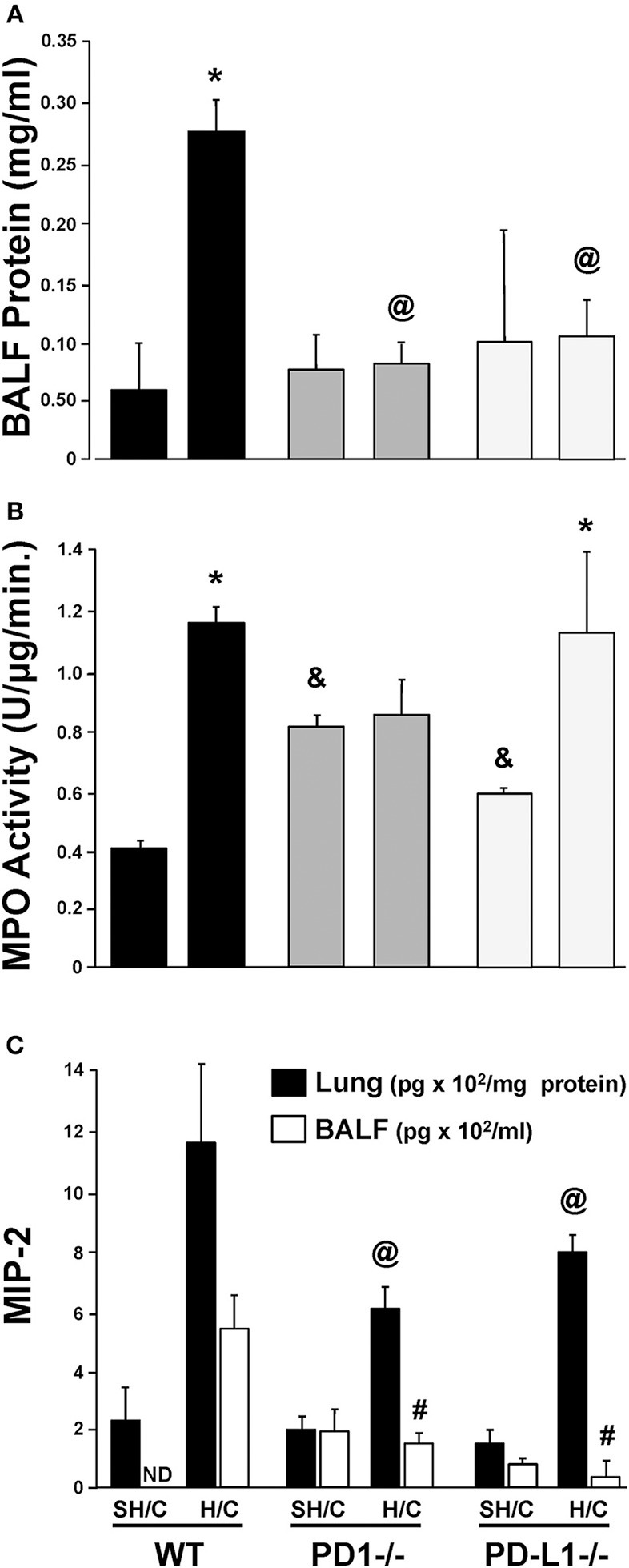
Concentration of protein in bronchial alveolar lavage fluid (BALF) as an assessment of lung injury in SH/C and H/C in PD-1^−/−^, PD-L1^−/−^ and WT Control mice **(A)**. *N* = 5–7/group; ^@^*p* < 0.05 vs. Control H/C; *p < 0.05 vs. respective SH/C. Lung tissue myeloperoxidase activity (MPO) in the lungs of PD-1^−/−^, PD-L1^−/−^ and WT Control mice following SH/C and H/C **(B)**. *N* = 3–5/group; *p < 0.05 vs. respective SH/C; ^&^*p* < 0.05 vs. Control SH/C; ^@^*p* < 0.05 vs. Control H/C. Neutrophil chemotactic protein, MIP-2, in lung tissue and BALF in those same groups **(C)**. Mean ± SEM, *N* = 5–7/group; ^@^*p* < 0.05 vs. Control H/C (lung protein); ^#^*p* < 0.05 vs. Control H/C (BALF). One-way ANOVA and a Tukey's multiple comparisons test.

Increased lung micro-vascular permeability is characteristic pathology in patients and in our experimental model of iARDS (20, 23). Bronchalveolar lavage fluid (BALF) protein leak was measured to assess loss of EC barrier function/lung tissue injury in Hem/CLP (H/C) animals' lungs compared to Sham Hem/CLP (SH/C) controls. Both PD-1^−/−^ and PD-L1^−/−^ mice had significantly reduced BALF protein levels compared to WT controls in response to H/C induced iARDS (Figure 3A).

### PD-1, but Not PD-L1, Gene Deficiency Suppresses the Rise in Lung MPO/ Neutrophil Influx

Neutrophil influx into lung tissue is a feature of ARDS pathology in both the patient population (28) and in our animal model (20, 23). Using MPO activity in the lung tissue to assess neutrophil influx (20, 23), we found that, as previously described, animals deficient in the PD-1 gene exhibited an attenuated rise in lung tissue MPO levels following H/C (4). In contrast, PD-L1^−/−^ H/C mouse lung MPO activity was elevated similar to WT H/C mice (Figure 3B).

### Neutrophil Chemokine Protein, MIP-2, Is Decreased in Lung Tissue and BALF in PD-L1 ^−/−^ as Well as PD-1^−/−^ Mice Following iARDS

While neutrophil influx into the lung (increased MPO activity) did not decrease in PD-L1^−/−^ mice when compared to PD-1^−/−^, MIP-2, in both lung tissue and BALF from PD-1^−/−^ and PD-L1^−/−^ mice, was significantly decreased compared WT H/C controls (Figure 3C).

### PD-L1 Gene Deficiency Suppresses Local Pulmonary Levels of Pro-Inflammatory Mediators Following iARDS

**Figure 4:**
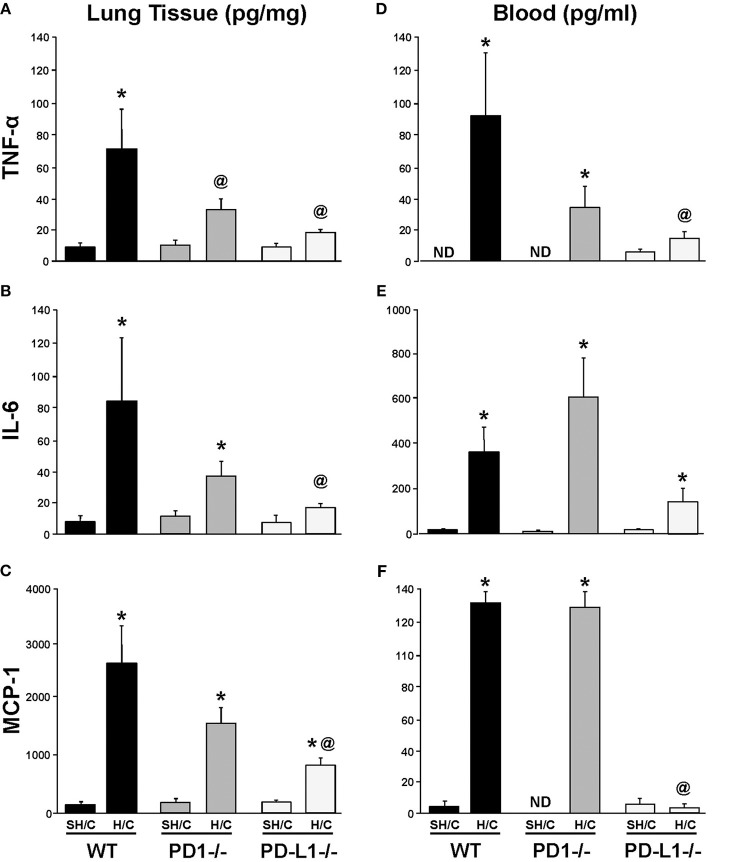
Tumor necrosis factor (TNF)-α, IL-6 and monocyte chemotactic protein (MCP-1) levels in lung tissue **(A–C)** and blood **(D–F)** of PD-1^−/−^, PD-L1^−/−^, and WT Control mice following SH/C and H/C. Mean±SEM, *N* = 5/group; *p < 0.05 vs. respective SH/C; ^@^*p* < 0.05 vs. WT Control H/C; ND, not detected. One-way ANOVA and a Tukey's multiple comparisons test.

The levels of lung TNF-α, IL-6, and MCP-1, were consistently elevated in WT H/C animals' lung tissue compared to the respective SH/C group. Only mice deficient in PD-L1 produced a consistent marked suppression of these mediators, while attenuation noted with the PD-1^−/−^ H/C mice was less consistent from a statistical perspective (Figures 4A–C). This difference between PD-L1^−/−^ and PD-1^−/−^ was even more overt when looking at the systemic levels of these same mediators in the blood/plasma, where again PD-1 gene deficiency had little to no impact on H/C induced rise in cytokines/chemokines, but lack of PD-L1 had marked impact (suppression) on the elevation of these agents (Figures 4D–F).

### PD-L1 Gene Deficiency Significantly Potentiates Expression of ICAM-1 Protein, on iARDS Mouse Lung Endothelial Cells

**Figure 5:**
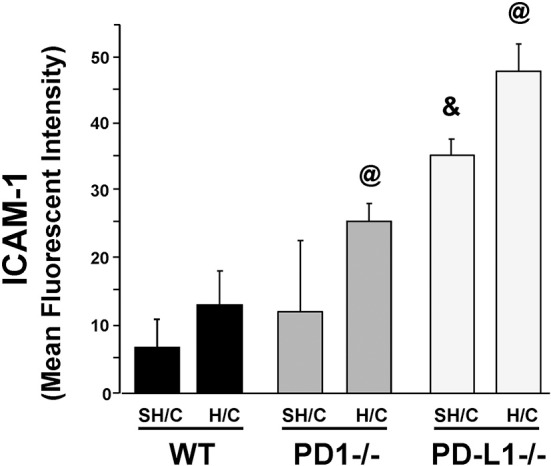
ICAM-1 expression on CD31^+^ ECs isolated from lung tissue of PD-1^−/−^, PD-L1^−/−^, and WT Control mice following SH/C and H/C. Mean ± SEM, *N* = 8/group; ^@^*p* < 0.05 vs. Control H/C; *p < 0.05 vs. respective SH/C. One-way ANOVA and a Tukey's Multiple comparisons test.

Since ICAM-1 is an EC surface protein that mediates firm adhesion of neutrophils to the activated endothelium (29, 30), we wondered if changes in EC ICAM-1 expression following H/C might be associated with the difference in neutrophil recruitment (MPO; Figure 3B) seen in the PD-L1^−/−^ animals compared to PD-1^−/−^. Lung tissue EC expression of ICAM-1 increased significantly in the PD-L1^−/−^ SH/C verses H/C groups as well as compared to corresponding WT controls (Figure 5). While PD-1^−/−^ Hem/CLP ICAM-1 was increased compared to H/C WT control, no significant difference was detected between WT SH/C and H/C groups (Figure 5).

### Deficiency in Either PD-1 or PD-L1 Gene Expression Attenuates the Marked Rise in the EC Growth Factor, Angiopoietin (Ang)-2 (but Not Ang-1), as Well as the Changes in Signaling Through Tie-2 Seen Following iARDS

**Figure 6:**
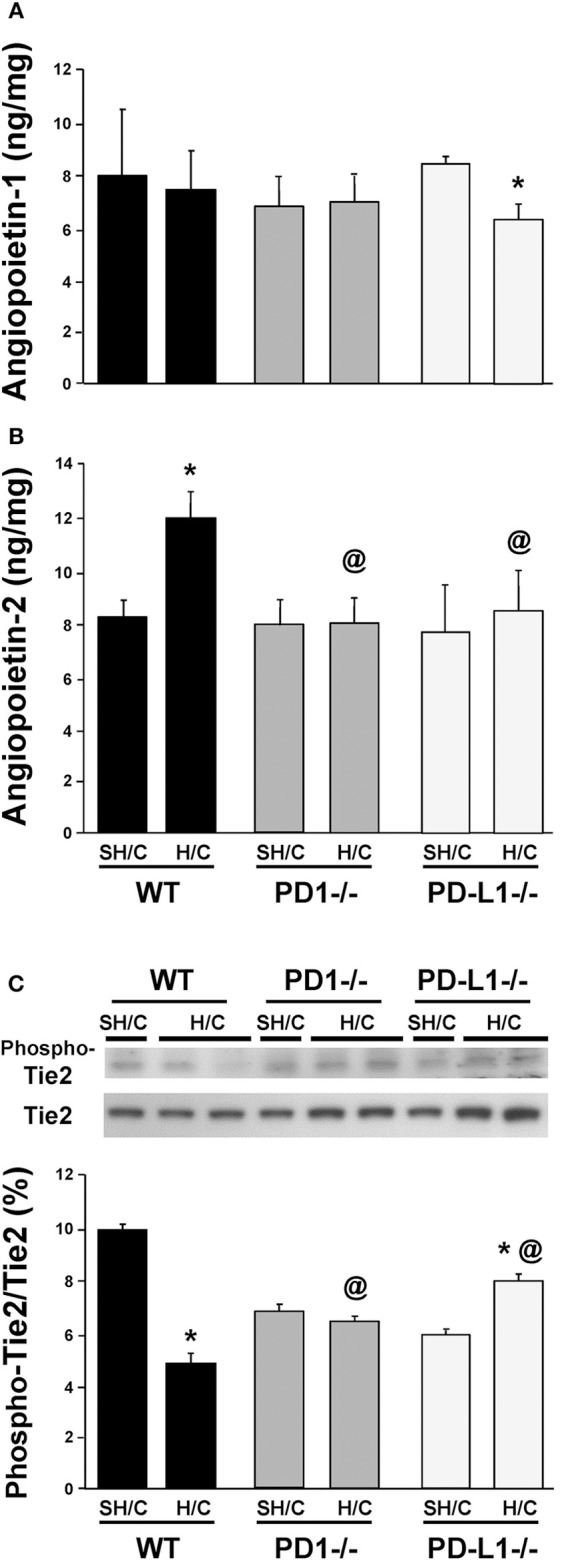
Endothelial growth factors Angiopoietin 1 **(A)** & Angiopoietin-2 **(B)** levels and the change in phosphorylated Tie2 (Phospho-Tie2) as percentage of total-Tie2 (Tie2) **(C)** in lung tissue from PD-1^−/−^, PD-L1^−/−^ and WT Control mice following SH/C and H/C. Mean ± SEM, *N* = 9/group; *p < 0.05 vs. respective SH/C; ^@^*p* < 0.05 vs. Control H/C. One-way ANOVA and a Tukey's Multiple comparisons test. Note, a typical western blot for Phospho-Tie2 and total Tie2 are provided in the upper half of section **(C)**.

Ang-2 is stored as a preformed entity, and when released from activated ECs, functions in an autocrine fashion, promoting vessel destabilization, inflammation and vascular permeability (31, 32). Ang-2 regulation of EC activation is counter-balanced by the paracrine release of Ang-1, largely derived from local pericytes, which acts to stabilize ECs and potentiate their survival via signaling through the tyrosine kinase receptor, Tie2, on ECs (33, 34). Specific to this, we have recently shown that blocking Ang-2 improves 10-day survival following H/C induced iARDS in WT mice (16). Here we observed, with the exception of the PD-L1^−/−^ H/C mouse group samples (which declined), that lung tissue Ang-1 protein levels typically did not change in WT and PD-1^−/−^ animals subjected to H/C when compared to SH/C group levels (Figure 6A). Alternatively, unlike WT H/C mice, where we observed a marked rise in lung tissue Ang-2 levels (corresponding with increased lung tissue injury) when compared to their respective SH/C group, Ang-2 protein levels in both the PD-1^−/−^ and PD-L1^−/−^ iARDS mouse lung tissue was significantly suppressed when compared to the WT H/C controls and no changes compared to their respective SH/C group (Figure 6B). Coincident with a decrease in Ang-2 protein expression in the lung of PD-1 and PD-L1 gene deficient H/C mice, we also noted that the marked decline in phosphorylated Tie2, as a percentage of total Tie2 expression levels, observed in WT H/C mice was attenuated in the H/C PD-1^−/−^ and PD-1L^−/−^ H/C mice (Figure 6C).

### Neutrophil Expression of PD-L1, Not PD-1, Is Potentiated Following Migration Into Lung Tissue in iARDS

We have previously shown that neutrophil/EC interactions play an important role in EC loss of (barrier) function in iARDS (16). In addition, we have shown that depletion of peripheral blood neutrophils prior to H/C significantly decreases indices of lung injury (decreased BALF protein) and EC activation (decreased Ang-2 protein) in our iARDS mice (16). In previous experiments in our lab, we have identified PD-1 expression on peripheral blood neutrophils (4) and in this study we characterized PD-1 expression on neutrophils that have trans-migrated into the lung following hemorrhage (Figures 1A,B).

**Figure 7:**
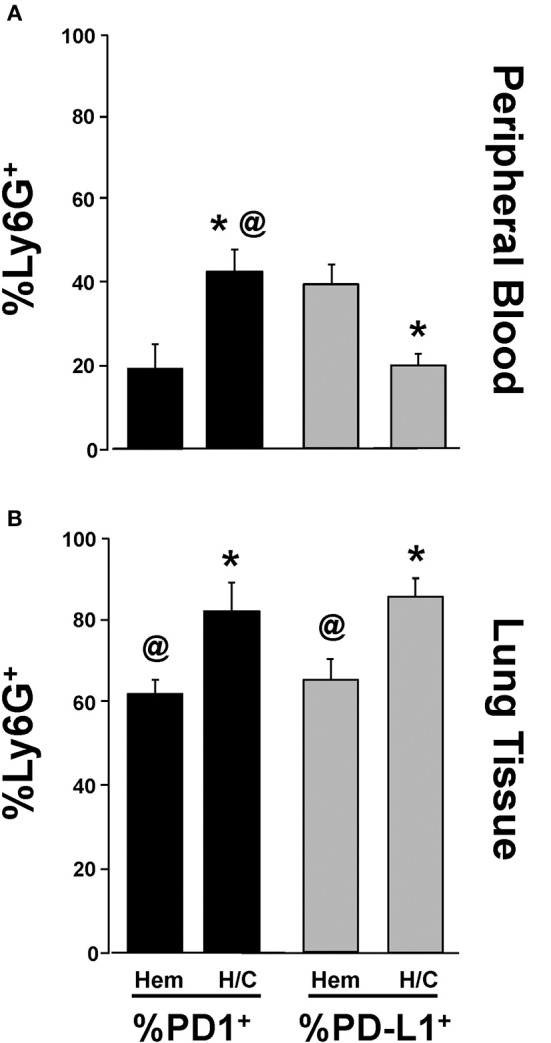
Comparison of changes in %Ly6G^+^ PD-1^+^ vs. %Ly6G^+^ PD-L1^+^ neutrophils in peripheral blood following Hem or H/C **(A)**. Comparison of changes in %Ly6G^+^ PD-1^+^ vs. %Ly6G^+^ PD-L1^+^ neutrophils in lung tissue following Hem or H/C **(B)**. Mean±SEM, *N* = 5–7/group. ^@^*p* < 0.05 vs. H/C %Ly6G^+^PD-L1^+^, *p < 0.05 vs. corresponding Hem).

The expression of PD-L1 on neutrophils has been reported by other laboratories in response to inflammation in autoimmune disease and infection (35, 36). However, to address the extent that PD-L1 expression on neutrophils might also be playing a role in the development of iARDS, we further investigated changes in PD-1 and PD-L1 expression on neutrophils immigrating into the lungs. To do this, we compared PD-1 and PD-L1 expression on peripheral blood neutrophils (Figure 7A) vs. neutrophils that had trans-migrated into the lungs (Figure 7B). The percentage of PD-1^+^Ly6G^+^ neutrophils increased in both peripheral blood and lung tissue following H/C when compared to hemorrhage (Hem) only (Figures 7A,B). However, while the percentage of PD-L1^+^Ly6G^+^ neutrophils decreased in peripheral blood following H/C, neutrophils that had transmigrated into the lung showed significantly increased frequency of cells expressing PD-L1 (Figures 7A,B).

### PD-L1 Gene Deficiency Differentially Regulates *ex vivo* EC Capacity to Release Angiopoietin-2 vs. Cytokine, IL-6, and the Neutrophil Chemokine, MIP-2

**Figure 8:**
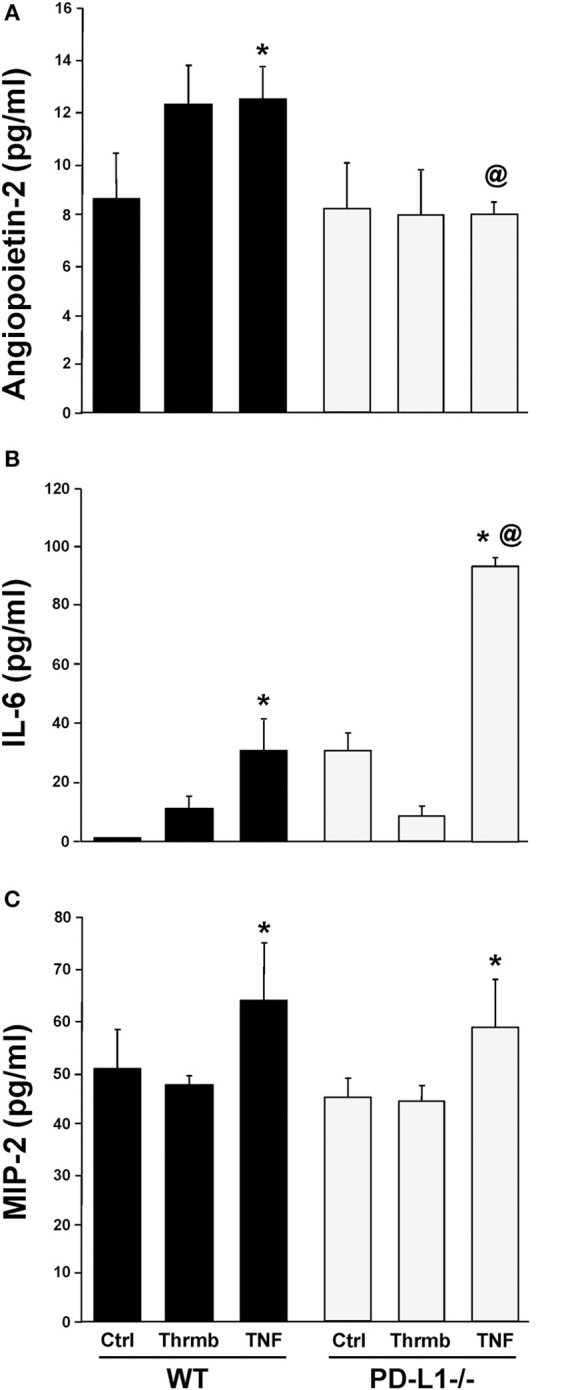
Endothelial cell release of Ang-2 **(A)**, IL-6 **(B)**, and MIP-2 **(C)** into culture supernatant from WT and PD-L1^−/−^ cell culture following a 4-h stimulation with either Thrombin (Thrmb) or TNF-α (TNF) or media Control (Ctrl). Mean±SEM, *N* = 5 mice/group/experiment, 3 experiments; *p < 0.05 vs. respective SH/C; ^@^*p* < 0.05 vs. Control H/C. One-way ANOVA and a Tukey's Multiple comparisons test.

Having found changes in indices associated with the development of iARDS in our model specific to the PD-L1^−/−^ phenotype, we further characterized lung ECs from PD-L1^−/−^ mice *in vitro*. ECs were isolated from WT or PD-L1 gene deficient mouse lungs, grown to confluence, and stimulated with either TNF-α (40 ng/ml), Thrombin (1 U/ml) to assess vascular injury verses inflammatory response, or culture media for Control. Of significance to our study here, neither TNF-α nor Thrombin treatment increased Ang-2 release from PD-L1^−/−^ ECs (Figure 8A). This matches our *in vivo* lung tissue findings from PD-L1^−/−^ H/C mice (Figure 6B). Also, consistent with H/C (iARDS) mouse lung tissue, increased MIP-2 was measured in the supernatant from isolated pulmonary PD-L1^−/−^ ECs following TNF-α stimulation (Figure 8C). Interestingly, while TNF-α stimulation increased IL-6 release by both WT and PD-L1^−/−^ ECs, supernatant IL-6 was significantly elevated by TNF-α treatment of PD-L1^−/−^ ECs (Figure 8B).

### PD-L1 Gene Deficiency Attenuates EC Induced Changes in Adherence Junction Protein Expression Seen in Response to iARDS

**Figure 9:**
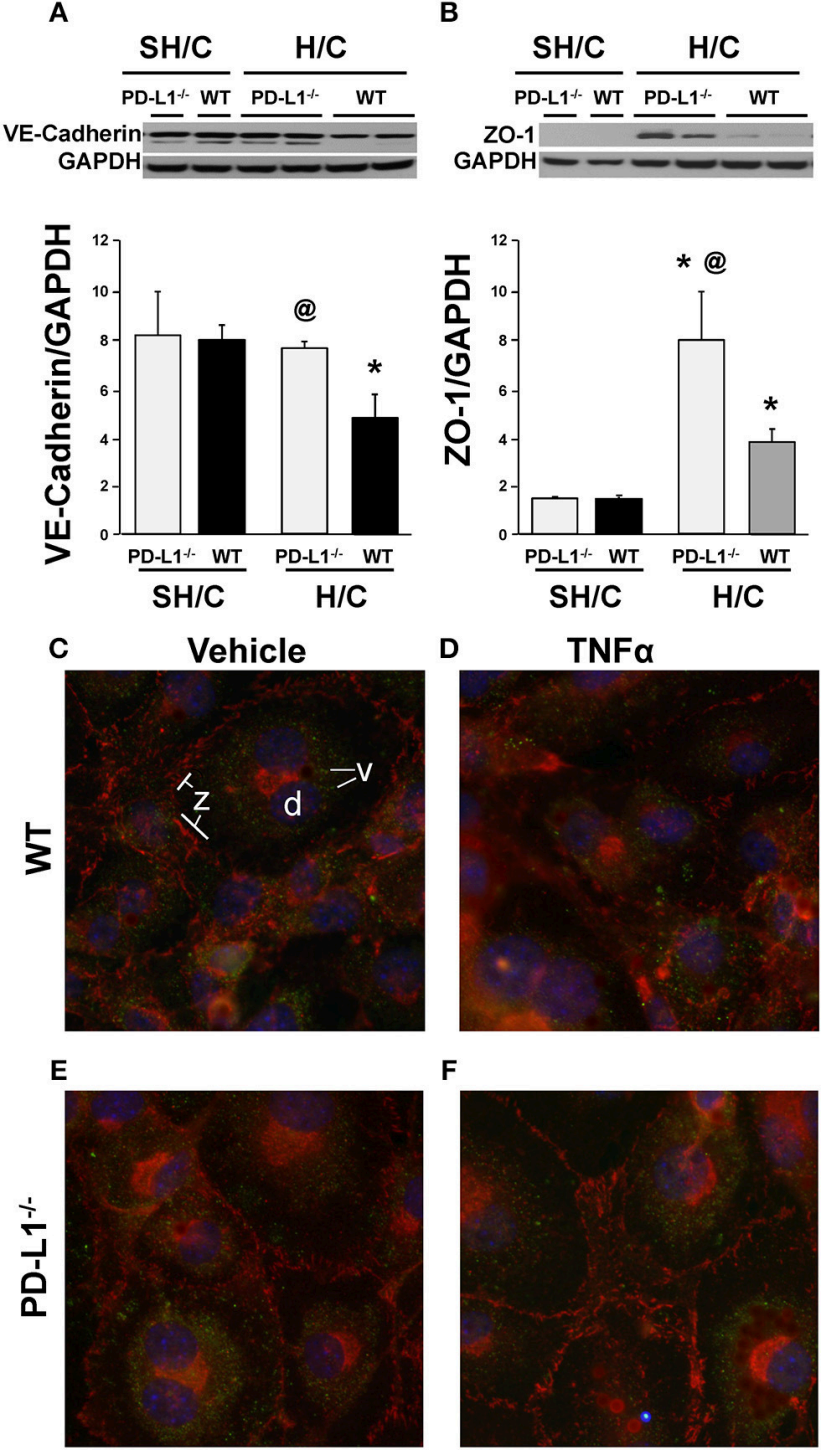
Western blot analysis of the changes in VE-cadherin **(A)**, ZO-1 **(B)** expression relative to GAPDH expression in ECs derived from WT or PD-L1^−/−^ mice subjected to SH/C or H/C. Mean ± SEM, *N* = 5 mice/group/experiment, 3 experiments, *p < 0.05 vs. respective SH/C, @*p* < 0.05 vs. Control H/C. One-way ANOVA and a Tukey's Multiple comparisons test. Note, a typical western blot for VE-cadherin or ZO-1 relative to total GAPDH are provided in the upper half of section **(A,B)**, respectively. Subsequently, EC monolayers, derived from WT **(C,D)** or PD-L1^−/−^ **(E,F)** naïve mice, were stimulated with media alone **(C,E)** or TNF-α **(D,F)** for 4-h and stained with antibody-fluorochrome conjugates against VE-cadherin (v) (Alexa 488; pseudo color green), ZO-1 (z)(phycoerythrin-PE; pseudo color red) as well as with DAPI **(D)** a nuclear counter stain (pseudo color blue), to examine the changes in the visual changes in cellular expression and location. A representative image (40x) for each treatment are provided (3 independent experiments).

To further investigate the potential mechanism(s) imparting improved iARDS survival in PD-L1 gene deficient mice, we examined changes in the expression of EC cell-cell adhesion protein, VE-cadherin, and an Occludins tight junction signaling protein, Zona Occludins (ZO)-1. The decrease in lung tissue VE-Cadherin observed following H/C in the WT mice was abrogated in the PD-L1^−/−^ H/C mice (Figure 9A). In addition, ZO-1 protein was significantly increased in PD-L1^−/−^ H/C lung ECs compared to WT H/C (Figure 9B).

To visualize these results, using fluorescence microscopy, we examined WT and PD-L1^−/−^ mouse derived lung EC monolayer expression of VE-cadherin and ZO-1 following 4-h stimulation with TNF-α or media. Expression of these proteins (Figure 9C), associated with maintenance of EC barrier functions, were decreased in TNF-α treated EC monolayers derived from WT mice (Figure 9D). In contrast, VE-cadherin expression on EC monolayers derived from PD-L1^−/−^ mice were similar to their non-stimulated controls (Figure 9C, WT and Figure 9E, PD-L1^−/−^) and ZO-1 staining appeared to increase (Figure 9F).

## Discussion

The ubiquitous expression of PD-1 on immune cells and PD-L1 on immune, as well as non-immune cell populations in many tissues and organs suggests a broader role for these molecules than the frequently reported T-cell immune regulatory responses. We have previously shown that PD-1 plays a role in potentiating the morbidity and mortality, associated with sepsis and iARDS, that we observe in our hemorrhagic shock (Hem) and/or CLP challenged mice (2, 4). Furthermore, our laboratory and other investigators have documented that in patients with severe septic shock, ARDS or severe injury (as determined by increased APACHE 2 scores), peripheral blood T-cells and monocytes exhibit a marked increase in PD-1 and PD-L1 expression (2, 8, 37–39). Importantly, in patients who have recently succumbed to sepsis, there is evidence of increased lung tissue PD-L1 expression at post-mortem assessment (40). However, as this clinical data is correlative in nature, in this study we employed our mouse model of hemorrhagic shock-induced priming for the development of iARDS following septic challenge to investigate the role of pulmonary PD-L1 and/or PD-1 expression in the development iARDS.

Our initial findings showed that following hemorrhage alone (Hem), expression of PD-1 and PD-L1 increased on Ly6G^+^ neutrophils (Figures 1A,B) and CD31^+^ lung cells, respectively (Figures 1C,D). Despite our previous observations that a significant influx of neutrophils in the lungs is not seen 24 h post-hemorrhage (20, 41), we were able to confirm an increase in Ly6G^+^ PD-1^+^ (neutrophils) expression between 6 and 24 h post-Hem (Figure 1E). Interestingly, PD-L1 expression on CD31^+^ ECs decreased overall (Figure 1F). This could potentially speak to the distinctive activation patterns of “priming” in these 2 cell populations and/or kinetics of PD-1/PD-L1 expression.

To better characterize the contribution of PD-L1 expression, the response of PD-L1^−/−^ mice were compared to that of PD-1^−/−^ or WT background animals in our model of H/C induced iARDS. Our initial question was whether PD-L1 gene expression, as we had previously shown for PD-1 gene deficiency (4), influenced iARDS mortality. Similar to PD-1 gene deficient animals (28), PD-L1 gene deficiency also provided a significant 10-day survival benefit to mice following iARDS (Figure 2). We next compared indices of lung injury and inflammation associated with iARDS in our model. Consistent with improved survival, a significant decrease in BALF protein (vascular permeability) was seen in both PD-1^−/−^ and PD-L1^−/−^ iARDS mice compared to WT controls (Figure 3A). However, this vascular leakage appears to be independent of neutrophil influx into lungs since deficiency of the PD-1 gene suppressed the rise in lung MPO activity, but no difference in MPO activity was observed between PD-L1^−/−^ and WT background control H/C mice (Figure 3B). In contrast to MPO data, neutrophil chemotactic protein, MIP-2, was significantly decreased in both lung tissue and BALF derived from PD-1^−/−^ as well as PD-L1^−/−^ H/C mice when compared to WT H/C animals (Figure 3C).

One possible factor that could account for unchecked neutrophil influx (MPO) in conjunction with significantly decreased neutrophil chemotactic protein, MIP-2, in PD-L1^−/−^ H/C mice may be the concurrent increase in ICAM-1expression on PD-L1^−/−^ H/C ECs (Figure 5). ICAM-1 expression on pulmonary vascular ECs typically increases during inflammation, serving to increase neutrophil adhesion and migration from the vasculature into the tissue (42, 43). Decreased ICAM-1 in the absence of neutrophil expression of PD-1 and conversely, increased ICAM-1 in the absence of PD-L1 gene expression on EC interacting neutrophils suggests that PD-L1 and/or signaling resulting from PD-1:PD-L1 ligation may play a role in maintenance/regulation of quiescent endothelium by decreasing neutrophil adherence and EC stimulation.

A decrease in neutrophil:EC interactions propagating the inflammatory response (and decreased vascular permeability) in PD-L1^−/−^ mice may also contribute to the general suppression of pro-inflammatory mediators. Local and systemic levels of TNF-α, IL-6 and MCP-1 decrease in PD-L1^−/−^ mice following H/C compared to PD-1^−/−^ H/C and WT control (Figures 4A–F). As we describe in Figure 1, the percentage of PD-1^+^ neutrophil and PD-L1^+^ lung ECs increase following hemorrhage as compared to Sham/Hem, potentially contributing to the dysregulated responsiveness or “priming” (sequestration and delayed apoptosis) of migrating neutrophils and loss of endothelial barrier function we see in our model following a subsequent septic challenge (19, 21). Loss of PD-1 expression may improve survival by contributing to the resolution of the inflammatory response while PD-L1 gene deficiency appears to more closely impact EC function, potentially associated with EC activation and Ang/Tie2 signaling pathway.

Endothelial growth factors, Angiopoietin-1 and -2 have been shown to regulate angiogenesis, cell survival and EC response to inflammatory mediators (16, 44–46). This occurs through regulated expression of Ang-1, Ang-2 and their binding to a shared receptor tyrosine kinase-2, Tie2, predominantly expressed on ECs (47, 48). Ang-2/Tie2 binding is associated with increased vascular permeability and decreased Tie2 phosphorylation, and has been reported to be significantly elevated in patients with ARDS and in our experimental model of iARDS (16). In this respect, as we have recently shown that the release of Ang-2 detected in animals subjected to H/C (as well as the associated rise in lung BALF protein leak) appears to be affected by pulmonary EC/ neutrophil interaction (16). We hypothesized that the expression of the either the PD-1 and/or the PD-L1 gene product could alter these important EC growth factors involved in maintaining vascular function. To assess PD-L1 and PD-1's potential contribution to Ang-1:Ang-2/Tie2 mediated endothelial activation in response to iARDS, we measured protein levels of Ang-1 and Ang-2 in PD-1^−/−^ and PD-L1^−/−^ gene deficient mice following H/C induced iARDS. While Ang-1 levels in both the WT background control H/C and the PD-1^−/−^ H/C mice decreased (when compared to their respective SH/C controls) there was a small but statistically significant increase in the PD-L1^−/−^ H/C mouse Ang-1 levels (Figure 6A). However, deficiency in either PD-1 or PD-L1 markedly attenuated the rise in lung tissue Ang-2 levels (Figure 6B), while restoring the downstream signaling via Tie2 (phosphorylated-Tie2 as a percentage of total Tie2) that was lost in WT H/C mice (again, as compared to each groups respective SH/C controls)(Figure 6C). This data implies that the effects of PD-1:PD-L1 interaction/signaling on the Ang/Tie2 pathway are more likely directed at the regulation of EC activation. Relative to EC activation through the Ang/Tie2 pathway, pericytes on the abluminal surface of vascular ECs contribute significantly to the modulation of EC activation through the release of Ang-1 (49). Pericytes may be contributing indirectly to the survival benefit seen for PD-L1^−/−^ iARDS mice here, as the anti-inflammatory/vascular stabilizing EC phenotype associated with Ang-1 was not decreased in PD-L1^−/−^ H/C lung tissue compared to PD-1^−/−^ or WT H/C. How the pericyte's capacity to release Ang-1 might be affected by H/C is not well-understood, but it may be related to neutrophil/EC interactions driving pericyte dissociation from activated endothelium and the subsequent decrease in Ang-1 available to bind to Tie2. We have shown that neutrophil depletion prior to H/C restores Ang-1 expression to SH/C levels (19), similarly reducing loss of function.

Our laboratory has reported lower blood leukocyte PD-1 expression associated with improved patient survival from ARDS (4). Alternatively, PD-L1 has been shown to be over-expressed by neutrophils in the blood of patients with active tuberculosis infection (35) and HIV/AIDS (36); thus, suggesting that increased PD-1 and/or PD-L1 expression may be associated with a failure to control disease pathology. A comparison of PD-1 vs. PD-L1 expression on peripheral blood neutrophils vs. neutrophils that had trans-migrated into lung tissue showed that %PD-1^+^Ly6G^+^ expression increased in both peripheral blood and lung tissue following H/C compared to WT controls. However, while percentage of PD-L1^+^Ly6G^+^ cells decreased in peripheral blood following H/C (Figure 7A), this trend was reversed on the trans-migrated neutrophils (Figure 7B). Interestingly, an increase in the total % PD-1^+^Ly6G^+^ and %PD-L1^+^Ly6G^+^ cells was seen in lung tissue vs. blood. While the functional significance of these changes in PD-1/PD-L1 expression on these neutrophil populations has yet to be fully elucidated, these data imply that the trans-migrated neutrophil, which appears in the injured lung in response to H/C induced iARDS, may be the result of either a selective recruitment of a given sub-population of neutrophils (PD-1^+^ vs. PD-L1^+^) to actively diapedesis or alternatively, expression of a differentiating phenotype that appears as a result of specific PD-1:PD-L1 interactions during diapedesis (neutrophil/EC) and/or interactions occurring within the lung microenvironment.

With this stated, as PD-L1 is not known to possess the same type of signaling motifs, e.g., ITIM, ITSM, SHP and/or GB2 motifs, as seen with PD-1 and other related members of this co-inhibitor family. One would therefore speculate that recruitment/association of a novel signaling protein(s) is required. However, how this might occur is unclear at present, and beyond the scope of this study, but cross-linking PD-L1 has (independent of signaling mediated by PD-1) been recently shown to not only potentiate the process of apoptotic cell death in the PD-L1 expressing target cells, but such ligation also up-regulated interferon production (50).

To better characterize the potential contribution(s) of PD-L1 to Ang-2/Tie2 regulation of EC barrier function, we compared isolated lung ECs from WT and PD-L1^−/−^ mice in *in vitro* cell culture. Supernatants collected from EC monolayers following stimulation with Thrombin or TNF-α showed that PD-L1 gene deficiency did not impact EC release of neutrophil chemokine, MIP-2 (Figure 8C). This is consistent with *in vivo* findings showing no change in lung tissue MPO (neutrophil influx to lung) (Figure 3B). In contrast to *in vivo* lung tissue and plasma data, a significant increase in IL-6 was measure in PD-L1^−/−^ EC supernatant compared to WT (Figure 8B), this may be a result of the isolated culture environment and lack of signaling from cells typically associated with the endothelium. Ang-2 release, similar to our findings in lung tissue from H/C iARDS mice, was significantly reduced in stimulated PD-L1^−/−^ ECs (Figure 8A).

Immuno-fluorescent microscopy and Western Blot analysis of junction proteins associated with EC barrier integrity provide further data supporting the survival benefit seen for PD-L1^−/−^ iARDS mice. VE-cadherin is an adherens junction protein important in maintaining EC barrier function. Loss of VE-cadherin expression is associated with increased vascular permeability (51). ZO-1 is a protein associated with the occludins tight junction family (52). Both of these proteins complex with the actin cytoskeleton maintaining EC junctional integrity. EC monolayers from PD-L1^−/−^ mice show no loss of VE-cadherin or ZO-1 expression following TNF-α stimulation when compared to WT ECs (Figures 9A,B). Western Blot analyses of cells harvested from non-fixed monolayers confirm these findings (Figures 9C,D). This *in vitro* cell culture data further suggest that PD-L1 expression may contribute to downstream EC activation/reprogramming, EC:neutrophil interactions, altered release of Ang-2 and the loss of vascular barrier integrity.

Taken together, these findings also imply that PD-1:PD-L1 interactions have a potential role(s) in not only leukocyte:leukocyte, but leukocyte:non-immune cell interactions in the pathogenesis of experimental iARDS; presenting potential novel therapeutic targets in the treatment of ARDS.

## Author Contributions

JL-N designed research and experiments, and wrote manuscript. JL-N and XH performed research and data analysis. SM, EF, C-SC and AA performed data analysis and contributed to manuscript writing, editing and revision.

### Conflict of Interest Statement

The authors declare that the research was conducted in the absence of any commercial or financial relationships that could be construed as a potential conflict of interest.

## Abbreviations

Ang-1: Angiopoietin-1
Ang-2: Angiopoietin-2
BALF: bronchoalveolar lavage fluid
CLP: cecal ligation and puncture
EC(s): endothelial cell(s)
H: hour(s)
Hem: hemorrhage alone
H/C: our mouse model of hemorrhagic shock (Hem) induced “priming” followed by the induction of sepsis (CLP) for the development of iARDS
HIV/AIDS: human immunodeficiency virus/acquired immunodeficiency syndrome
iARDS: indirect acute respiratory distress syndrome
ICAM-1: intracellular adhesion molecule 1
IL-6: interleukin-6
IV: intravenous
MCP-1: monocyte chemotaxin protein-1
MIP-2: macrophage inflammatory protein-2
MPO: myeloperoxidase
PD-1, Programmed cell death receptor-1 (mice deficient in this gene: PD-1^−/−^)
PD-L1, Programmed cell death receptor ligand-1 (mice deficient in this gene: PD-L1^−/−^)
siRNA: short interference ribonucleic acid
TICU: trauma intensive care unit
Tie2: –tunica intima endothelial receptor tyrosine kinase-2
TNF-α: -tumor necrosis factor-alpha
WT: wild type (background mouse strain)
ZO-1: – Zona Occludins-1.
